# Therapeutics of Heart Disease

**Published:** 1895-10

**Authors:** Horatio C. Wood

**Affiliations:** Philadelphia, Pa.


### THERAPEUTICS OF HEART DISEASE.*

BY HORATIO C. WOOD, M.D., LL.D.,
PHILADELPHIA, PA.

I want to talk to you concerning a disease which, as you know, is everywhere prevalent throughout the civilized world. And I propose first to carry you back to the days of my childhood, and one of my first patients (an old lady probably eighty-five years of age), who taught me this great lesson: that it is possible, with a very small amount of valvular integrity, to pass through a long life of labor and usefulness.

Now, when we come to the why of this thing, we can go back to our early childhood days and remember how, in the frosty mornings, we crawled out of bed and ran to the old pump under which we washed ourselves. We had to redouble our strokes in order to keep warm. And this is what the heart does, and it is what we call compensatory hypertrophy. Almost all your practical skill in treating a disease of the heart must rest upon practical skill in deciding, not whether this valve is diseased or that valve is diseased, but whether the increased power of the heart has or has not kept pace with the increased work required of the heart.

And here I want to say a word in regard to this question of diagnosis. There are some who think that when the aortic valve is diseased you should never give heart tonics or heart stimulants; that digitalis is not indicated. Not so, gentlemen. The aortic valve is no different in its functions and its relations to drugs than is the mitral valve. It so chances that the mitral disease is produced rapidly during youth, and the aortic disease comes on slowly during age; and in the one case the heart is more prone to be unable to adjust itself to the new conditions, and you get cardiac weakness.

It is not a question whether this valve be diseased or whether that valve be diseased, but is a question of the relation between the work and the power.

We want, then, always in every case of heart disease, from the

*Abstract of address before the Cleveland Medical Society, June 28, 1895.

very beginning to guard against this failure of development of compensatory hypertrophy. But before taking up the subject of drugs and their uses in failing heart, let me say a word to you in regard to the effects of failure of heart power. Where does the strain come from, or where is the lack apparent? When the heart fails the blood fails to be in the aorta and its tributary vessels; and when the aorta fails and its tributaries are empty the veins are full, and consequently you may take for a sign of failing heart that there is over-venous repletion of the skin, congestion of the lungs and liver, and edema of the extremities.

Now I want to call attention to this fact, that it is the liver among all organs, next to the lung at least, that feels this excess of blood in the venous system. Engorgement of the portal circulation is present almost always in every case of heart disease. It is largely for this reason that mercurials are of the value that they are in heart disease. And if I have had any special success in the treatment of this class of cases, it is because I have recognized the value of mercurials. Mercurial purges and corrosive sublimate given in long-continued small doses are of the greatest importance. The fiftieth of a grain, or the sixtieth, or even one-hundredth of a grain of corrosive sublimate, given with the tincture of the chloride of iron, will sometimes effect almost a revolution, aiding your true heart tonics in the most remarkable manner; aiding, so to speak, in the digestion and absorption of the medicine.

I want here also to force upon your attention the necessity of cardiac rest. There has grown up a wide school of therapeutists in Germany who teach that we are to cure heart disease by cardiac gymnastics, climbing mountains, etc. Gentlemen, the heart is an organ that never rests under any circumstances, and do you believe it is common-sense teaching that you can take an organ that has had no rest and is exhausted and build it up by piling on to the load which nature has put upon it? Not so. In the German mountain cures it is not the extra work put upon the heart that cures, but it is the extra work put upon the muscles of the body that cures. Such cases are not instances of genuine failing heart.

Another set of cases are cured by the mountain treatment. These are the cases with a heart working irregularly because the blood is loaded with uric acid derivatives, or uric acid-like compounds.

But you take a spare American with bad heart and you put him up the mountains, and you ought to put the lid on the coffin so he could carry it up and bury himself.

Leaving these means, which seem to me of great practical importance, I come to ask your attention to the drugs we have to use in cases of failing heart. I have studied adonidine, cactus, convallaria, and other of the newer remedies, and I do not think they have real value. I have never seen myself any good result obtained from any cardiac drug whatever that could not be obtained from nitroglycerine, strophanthus, and digitalis; and I never had any satisfaction whatever in the treatment of real downright heart trouble with any other cardiac drugs than these three.

First, nitroglycerine. Nitroglycerine lays itself aside from the other two cardiacs or cardiac drugs in the fact that it dilates arterioles and lowers arterial pressure. More than that: nitroglycerine probably has a powerful momentary stimulant influence on the heart-muscle, but if one passes over in the slightest degree the dose, the stimulant action passes immediately into one of immense depression. Kill the animal with nitroglycerine, and its heart is relaxed as a wet paper bag; kill the animal with strophanthus or digitalis, and its heart is spasmodically contracted.

Then, again, remember that nitroglycerine, like prussic acid, acts only for a few minutes. The profession has been giving prussic acid for years three or four times a day. Now it is a well known fact that if a man takes a fatal dose of the acid and survives thirty minutes he almost invariably gets well. All symptoms are usually gone in twenty minutes. A fatal dose may show no influence after twenty minutes. How much influence will there be in two hours from a dose that at no time has caused any demonstrable effect? Now it is very nearly so with nitroglycerine, and therefore, if you are going to use nitroglycerine at all, use it in small doses and at very short intervals. It is only valuable as a momentary pick-me-up to the heart. It is only useful in the crisis of the attack, and is especially efficient if the attack takes the form of angina pectoris. How it acts I do not know. It is very possible it may be by relaxing spasm, rather than by stimulating the heart.

And now let me call your attention to strophanthus. Strophanthus is a drug which is used in the chase. I believe it is true that every drug used in the chase of animals by savage peoples is one that acts either upon the nerve or muscle, the reason being that the man who chases an animal wants to get him, and wants to use something that will stop him from running away. Strophanthus is a muscle poison. It only acts upon the heart as it acts upon the other muscles, but it so happens that in man the heart muscle is more susceptible to its influence than are the voluntary muscles; and so in human medicine we are able to get a stimulant effect on the heart before we get it on the other muscles. It is a drug, therefore, that acts directly as a stimulant to the heart muscle. But there is no reason to think that, like digitalis, it acts further as a tonic than as a stimulant. Then, again, the muscle of the arteries is acted upon by strophanthus, and so it contracts the arteries. It increases arterial pressure, it empties the veins and fills the arteries. It differs further in its action from digitalis in being more distinctly diuretic; it is much more prompt than digitalis, acting at once; it is much less permanent than digitalis.

Let us now study digitalis, which always in proper doses elevates the arterial pressure. How? It does it, in the first place, by contracting the arterioles. It narrows the blood-paths, it lessens the amount of blood-space to be filled. It does this in a twofold way: by stimulating the vaso-motor center in the medulla; by acting on the arterioles themselves. Take a terrapin, cut out its nervous system, leave its heart intact; or cut out its heart, then put your fluid under pressure into the arterial system and have it come out from the vena cava. Add a little digitalis and it almost arrests the flow. It contracts the capillaries directly. But it acts upon the heart more powerfully. Every one knows the full, strong beat we can get from the drug. Especially do not forget that it stimulates the pneumogastric nerves as well as the heart muscle. It lengthens the interval between the beats. When that beat comes it is a great, mighty throb of blood. But digitalis is more than a stimulant to the heart. You take a heart which is beating one hundred and ten to one hundred and twenty times a minute: the veins are everywhere full, the aorta is empty. The diastole has not been long enough for the heart to expand and receive the blood. The heart is continually irritated by impulses coming up from every part of the body crying, Give us more blood. You give that heart digitalis. You quiet it, you take off its nervousness, you get the long diastole, you get the powerful systole; so there comes a great wave of blood through the arteries. In the failing heart the coronary artery gets little or no blood. At the very time when the heart is being overworked and overworried it is starving. But when the great wave of digitalis action comes, it swells out the aorta, it fills the coronary artery, it goes into every part of the heart, it brings sustenance and food. The old effete material that has been clogging the heart walls is all squeezed out by the powerful contraction of the muscle. Thus digitalis acts as a heart tonic, but it does more than this.

Digitalis has the power of stimulating to a point of intense activity the pneumogastric nerves. Expose a frog's heart, give it digitalis, and you will see the strange fight for mastery between the irritated pneumogastric nerve and the irritated heart muscle. Sometimes the digitalis arrests the frog's heart in diastole. It never does this after section of the pneumogastric nerve. It is when the pneumogastric nerve gets victory over systole that there is arrest in dilatation. Now don't you see how digitalis acts in failing heart? Not simply as a stimulant, but also as a tonic. It brings food to the heart at the time of its starvation and overwork; it squeezes out of the heart muscle the effete matters which have been lying there; especially does it stimulate the pneumogastric nerve, the trophic nerve of the heart, enabling it not only to quiet the heart into long diastoles, but to hasten during these periods the upbuilding of the heart structure.

Every now and then in our hospitals we see a case of heart disease in a poor man who has had no rest and no medical treatment; the heart seems hopelessly feeble, but a short course of digitalis brings not only immediate relief, but seems to lift the whole man up to a higher plane. It is because the digitalis has really helped the trophic nerve to use the food which has been given to the heart by the use of digitalis to the restoration of the structure, which was almost destroyed.

Before applying these considerations to individual cases, let me say a word or two about the preparations of digitalis. It does not make any difference which preparation, provided it has been made from a good drug. I speak now of the officinal preparations of digitalis. Digitalis is always a doubtful theme. Of two specimens one will be soluble, one insoluble; one is called the French, the other German. Digitalin is not of the nature of the active principle. It is a mere extract of digitalis more or less purified. There is a widespread belief that the infusion of digitalis is better than the tincture. Not so, gentlemen. The reason of the belief is that proportionate doses are not used. Commonly, digitalis tincture is given in five to ten-drop doses, the infusion from a teaspoonful to a dessertspoonful, which is equivalent to about twenty to forty drops of the tincture. Results are obtained from the infusion because it is given in larger doses.

Sometimes you will find a stomach that the infusion will agree with better than the tincture; sometimes one that the tincture agrees with better than the infusion. Practically there is no difference. Again, the tincture of digitalis lends itself fairly well to hypodermatic medication; infusion you cannot use.

I do not propose to occupy your attention at all with discussing the ordinary use of digitalis in heart disease. Only to call your attention to certain points. First let me say a word in regard to its employment in acute endocarditis, acute heart disease. Early in the case digitalis is very rarely indicated. It is more apt to do harm than good. The heart is already in a state of irritation. There are some cases of endocarditis—septic or malignant endocarditis—in which it does not make much difference what you give. But in ordinary rheumatic endocarditis, digitalis does harm. The heart is not weak but over-irritated. Usually tincture of aconite and similar drugs are what you want to try. But when the storm has gone by, the only salvation for the stricken child is the upbuilding of that heart into a great powerful organ that shall enable it to overcome the leak that has been left in the valve. There is no power to repair that valve. There is no rag that we can push or stick into that broken space. There must be increase of power, and so under these circumstances, so soon as the acute disease has passed by, it is absolutely important to begin the use of digitalis in small doses continually, given with great watchfulness. Recollect here, as in other cases, that when you give digitalis you give a drug which has a persistent influence. The moment you get the slightest effect that moment you stop the drug, for you know that effect will last hours, perchance days.

Passing by the ordinary use of digitalis, let me call your attention to its administration in large doses. And here I beg of you not to misunderstand me; I do not mean to say that the doses of digitalis I am going to speak of are to be used in the ordinary cases of heart disease. But there come times in the life of almost every case of heart disease when the heart fails to respond at all to the moderate dose of digitalis, and when the large dose of digitalis will have a most pronounced and beneficial effect upon it.

[The speaker here described some cases in which he had given forty to sixty drops of the tincture every three or four hours in order to get desired effects.]

A few words in regard to the so-called cumulative action of digitalis. Digitalis may be given through a length of time and suddenly there comes an explosion of its action.

This cumulative influence comes on at certain times: First place, when the drug fails to act upon the kidneys. Second place, when it is already in the body. You have a case of dropsy; you tap—reduce the pressure. Instantly the blood vessels take up the serum; that serum is loaded with digitalis. Then comes digitalis poisoning. It is not the drug that is in the body, but the drug that is in the blood that affects the heart.

A practical induction is: When you expect to tap a man suffering from heart disease or from any form of dropsy in which you have been giving digitalis, cease your digitalis for a length of time before you tap him.

Years ago I noted one class of seemingly incomprehensible cases; cases of mitral valve insufficiency, in which the heart seemed to be in the last stage of weakness; cases where I said to myself, "This man will be picked right up by digitalis." But when I gave the digitalis there came increased heart dullness, and an anginous horror that instantly demanded the withdrawal of the drug. I was sure that I was right in my diagnosis that the heart was weak and failing. I was sure that I was right in my physiology that digitalis was a heart tonic and stimulant. But when I put the two things together they did not work. The cases always went from bad to worse. At length I worked out the problem. The cases were, all of them, cases of mitral insufficiency, and they were really cases of exceedingly weakened auricle; an auricle that was toned down and thinned out until it was little more than the thickness of paper. Now, under the influence of digitalis there came back a reflex-wave through the mitral valve and that met the blood pouring in from the pulmonary veins. The thin, paper-like auricle could not stand the double pressure. It could not stand the strain of the blood pouring in and the blood pushing back through the insufficient mitral valve by strengthened systole. Whenever this condition exists nothing can be done. It is the shadow of death, and death very near at hand.

Digitalis has been much used in treatment of aneurysm. Gentlemen, it is death in aneurysm. The reason surgeons have not killed more cases of aneurysm is because they do not use digitalis in large enough doses to have any effect. Digitalis is the most dangerous drug known in aneurysm. You give a certain amount of strychnine, or a certain amount of atropine and get, let us suppose, twenty per cent. of increase of arterial pressure, but the pulse wave is small. Digitalis puts up the arterial pressure twenty per cent., but it does more than this. It makes a long diastole. It makes a great wave of blood; not a little tiny thread, but a great mass of blood rushing with full force down the arteries, coming into the chamber enlarged by atheromatous degeneration, stretching and tearing everything before it. It is the immense distension due to the large blood wave under the influence of digitalis which makes the drug especially dangerous. This is not merely theory. Some years ago a man was picked up in the street and brought into the hospital. He had a pulse you could scarcely feel; temperature four or five degrees below normal. I ordered digitalis to be freely given. At my visit the next day the man was sitting up enjoying himself, talking and laughing. I put my hand on his pulse and I got the tremendous big strokes of digitalis. As I was directing that the digitalis should be stopped, the man sprang with a great cry up into the air; there came a crimson flood from the mouth and nose, and the man dropped back dead. The man had an aortic aneurysm; we had ruptured it with our digitalis. At the autopsy the aneurysm was found torn right across. Gentlemen, when you have an aneurysm to deal with, don't use digitalis.

Let me speak of the contrast between digitalis and veratrum viride in the treatment of pneumonia. You have a case of pneumonia in the first stage. You give veratrum and you lower the heart action. More than that, you open out every blood vessel in the body. Here is the blood in that lung in excess. You can put the whole of the blood of the man in the abdominal vessels. You take away the blood from the lungs and put the whole body upon one plane of dilated arterioles, so to speak. That is what veratrum viride does in the beginning of pneumonia.

If you have a lung in a man in which the general system is adynamic, you can get at the same result in a different way. You have general relaxation. Now you give ergot freely, which contracts the blood vessels and brings about the same result by contracting the blood vessels in the lung and the whole body.

Digitalis comes into use in pneumonia in an entirely different stage. How does that man die in advanced pneumonia? Very often from paralysis, exhaustion, or arrest of power of the right heart. What is the reason? Because the right heart, heated almost to death by the fever, has to force blood through paths narrowed by the pressure of the exudation upon them. You give digitalis; it stimulates that right heart. It does not cure the pneumonia but it keeps the right heart up to its work, and by and by the pneumonia subsides.

